# A Survey on Perceptions of Complementary and Alternative Medicine among Undergraduates in China

**DOI:** 10.1155/2020/9091051

**Published:** 2020-01-30

**Authors:** Hui Xie, Tianqing Sang, Wenting Li, Li Li, Yankun Gao, Wenli Qiu, Hongguang Zhou

**Affiliations:** ^1^Department of Oncology, Affiliated Hospital of Nanjing University of Chinese Medicine, Nanjing 210029, China; ^2^Institute of Oncology, The First Clinical Medical College, Nanjing University of Chinese Medicine, Nanjing 210046, China; ^3^School of Pharmacy, Nanjing Medical University, Nanjing 210029, China; ^4^Department of Radiology, Affiliated Hospital of Nanjing University of Chinese Medicine, Nanjing 210029, China

## Abstract

In recent years, complementary and alternative medicine (CAM) is more widely known and used globally. This study was the first to investigate undergraduates' attitude toward CAM, and influencing factors and barriers for students to use CAM. Students of five different grades in six universities of China were selected for this study from February to May 2019. First, the participants were divided into two groups based on their majors and fulfilled a previously validated 10-item CAM Health Belief Questionnaire (CHBQ) to evaluate their attitudes toward CAM. Second, the chi-square test was used to analyze the differences between the groups, and correlation analysis was conducted to investigate the relationship of the data between the two groups. Third, we used frequency analysis to identify the types that students wanted to study and the barriers to use CAM. The overall mean score of the CHBQ was 48.87 ± 8.594, which was higher than that in other countries. The students in lower grades had a stronger desire to learn CAM than those in higher grades (89% vs 83%, *p* < 0.05). “Too time-consuming and bad tastes,” “Western medicine was enough,” and “lack of relevant knowledge” were found to be the main interruptions for students to use CAM. 82.3% of students wanted CAM to be incorporated into the curriculum and desired to learn more about CAM. 72.3% of the students who had never learned CAM wanted to know more about CAM. 55.5% of the students were willing to recommend CAM. Most undergraduates desired to learn more about CAM. It is necessary to introduce or integrate CAM courses into the present curriculum, and it should be started in the lower grades. We hope this study can provide evidence for the authority in China to make appropriate changes and integrate CAM into the college curriculum.

## 1. Introduction

Complementary practices are healthcare interventions used together with conventional medical practice, while alternative health practices are considered to be an option for conventional medical practice [1]. Influenced greatly by social and cultural traditions, complementary and alternative medicine (CAM) plays an irreplaceable role in health care around the world. The proportion of people who were treated by CAM ranges from 70% to 90% in Italy, Germany, Canada, and France [1]. In 2007, up to 40% of adults have received CAM therapy in the United States [2].

Having been included in the college curriculum, CAM is now being taught in most medical schools worldwide because of its high utilization rate [3–6]. In the 1990s, CAM courses were incorporated into the undergraduate medical curriculum by 12 (75%) medical schools in Canada [7]. In the United States, between 1996 and 1999, the number of medical schools integrating CAM into their curricula increased from 46 to 75 of 125 schools [8]. Furthermore, both the Association of American Medical Colleges and the American Medical Association have been actively discussing the strategies of integrating CAM into the conventional curriculum [8]. A survey conducted in Japanese medical schools in 2001 reported that about 16 (20%) schools had integrated CAM into their curricula [9].

In China, the most popular alternative medicine is traditional Chinese medicine (TCM). As one mandatory part of medical education in China, TCM education started over ten years ago, and students in medical universities must attend over 200 didactic hours of TCM courses [10]. It was reported that 33% of the medical students in Hong Kong had used TCM at least once, and 85% had ever tried TCM therapy. The proportions of students who held positive, neutral, and negative attitude toward TCM were in 52%, 41%, and 6%, respectively [11, 12]. However, the abovementioned research was only conducted in one school, and the respondents were restricted to students of pharmacy and Chinese medicine.

Lots of studies indicate that not only students of clinical medicine but also those of other majors hope to take more CAM courses [13, 14]. Currently, few studies have addressed the attitudes of undergraduate students in mainland China toward CAM. Therefore, this survey aimed to (1) survey the students' attitude toward CAM, the influencing factors, proportion of CAM courses in the curriculum, and the use of CAM; (2) prove the reasons for the absence of CAM in the curriculum; and (3) gauge the students' willingness to learn CAM.

## 2. Methods

### 2.1. Population and Data Collection Procedure

This is a cross-sectional survey with data from questionnaires collected from February to May 2019 among 2292 undergraduates of six universities in mainland China. The survey was targeted at the first-, second-, third-, fourth-, and fifth-year undergraduate students in universities of Nanjing, China. Students of medical universities (MU) were from the Nanjing University of Chinese Medicine (*n* = 826) and Nanjing Medical University (*n* = 622). The students of nonmedical universities (NU), in which CAM courses were rarely taught, were from Nanjing Audit University (*n* = 123), the Nanjing University of Posts and Telecommunications (*n* = 225), Hohai University (*n* = 203), and Southeast University (155). The students of clinical medicine included the students of traditional Chinese medicine (TCM) and Western medicine (WM). The former had CAM courses, and the latter had courses of WM in their curriculum, and the proportion of CAM and WM didactic hours were different between the students of TCM and WM.

**Figure 1:**
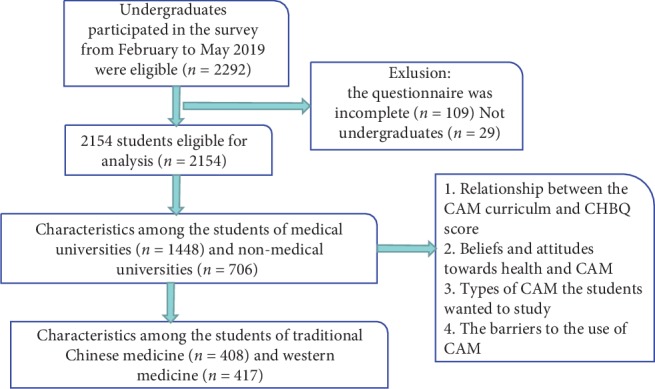
Flowchart of selecting study population.

The authors of this study were on the spot to answer any doubt that the students might have when they were filling in the questionnaire. All students attending the survey were voluntary. They were required to complete the questionnaire within 20 minutes, and then all questionnaires were collected. The inclusion and exclusion criteria and the procedure are shown in Figure 1.

### 2.2. Questionnaire

The questionnaire was based on questions used in previous surveys [15]: The CAM Health Belief Questionnaire (CHBQ) was translated from English to Chinese [8], and several items were added from a questionnaire designed by Hong Kong researchers [16]. The total score of the CHBQ is 70. A positive attitude to CAM was defined as a mean score exceeding the midpoint neutral score of 35. The students were allowed to select multiple options when answering questions labelled “multiple responses.” The definition of CAM was “medical and health systems, applications, and products currently not considered as part of conventional medicine” from the National Center for Complementary and Alternative Medicine (NCCAM). We also enlisted several types of CAM, including TCM, nutrition, acupuncture, spirituality/prayer, yoga, Tai Chi, Qi Gong, osteopathy, massage, chiropractic, homeopathy, meditation, and ayurveda medicine. To ensure the readability and apprehensibility of the questionnaire, we pretested 200 students and adjusted the questionnaire to make it more comprehensible.

### 2.3. Data Analysis

All data were incorporated into a Microsoft Excel spreadsheet and analyzed by SPSS version 20. Continuous variables including Likert scale questions were summarized using means and standard deviations, and categorical data were summarized using frequency distributions. Mann-Whitney *U* tests were implemented in the comparison of attitude score differences between the participants' categories, and chi-square test was used for categorical data between the groups. *p* < 0.05 was considered significant.

## 3. Results

### 3.1. Characteristics of the Participants

Table 1 illustrates the characteristics of the study population. The mean CHBQ score was 48.87 ± 8.594 in all students, indicating a positive attitude as it was above the arbitrary mid value of 35. The mean CHBQ score of MU was higher than that of NU. Gender, proportion of CAM courses, source of information, and self-application were the factors affecting the students' attitude toward CAM. However, there was no significant difference in grade, age, publicity for CAM in place of residence, and father or mother's level of education. The diagram showed that the majority of MU (48.6%) considered teachers as the primary source of CAM information, whereas for the NU, the main source of CAM information was media and Internet (41.4%). To verify the accuracy of the results, we conducted internal verification among the students of clinical medicine.

### 3.2. The Relationship between Proportion of CAM Courses and CHBQ Score

**Figure 2:**
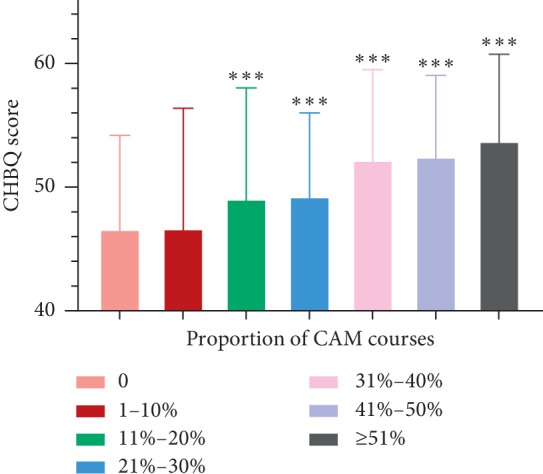
The relationship between the proportion of CAM courses and the CHBQ score. ^*∗∗∗*^*p* < 0.001 through comparison of each group with the group of ratio 0.

As shown in Figure 2, the proportion of CAM courses has a positive correlation with the CHBQ overall mean score in all students (Spearman *R* = 0.35). The attitude score was the highest when the proportion of CAM courses was over 51% (53.91 ± 7.305). The lowest attitude score appeared when the proportion of CAM courses was less than 10% (46.63 ± 7.57).

### 3.3. Beliefs and Attitudes toward Health and CAM

Over 79.1% students agreed that CAM should be integrated into WM (Table 3). Furthermore, nearly two-thirds (65%) of the participants believed that it would be better for clinical doctors to know CAM. Noticeably, 83.5 % of students disagreed that CAM was a threat to public health, which was contrary to a Malaysian study in which less than 25% of pharmacy students agreed to this statement [17]. In addition, 77.3% of students did not agree that CAM had no therapeutic but only placebo effect.

### 3.4. Types of CAM the Students Wanted to Study

Table 4 describes the types of CAM that the students wanted to study. The top three were massage (50.7%), acupuncture (48.6%), and TCM (47.3%), which was partly similar to the CAM types that Irish students were most interested in: massage and acupuncture [18]. In this study, the students showed little interest in homeopathy, Ayurveda medicine, naturopathy, and osteopathic medicine. This might be attributed to the fact that massage, acupuncture, and TCM were the best-known therapies in China. Therefore, most students claimed that they knew at least something about these types of CAM. Only a few students claimed they knew about chiropractic, meditation, Ayurveda medicine, homeopathy, and naturopathy, and many had never heard of these therapies before.

### 3.5. The barriers to the Use of CAM

**Figure 3:**
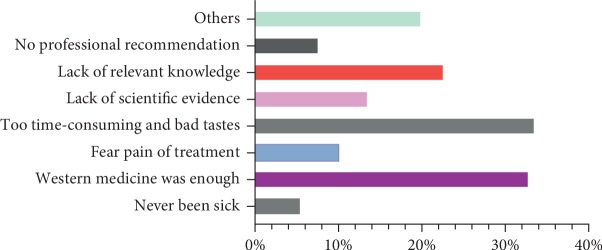
The barriers to the use of CAM.

Figure 3 reveals that 33.6% of students thought CAM treatment was too time-consuming and related it to bad tastes. About 32.9% of students thought that Western medicine was enough. About 22.7% of students admitted they did not know CAM well. Unlike surveys in other Asian countries, the students thought that lack of scientific evidence, information, and trained professionals were the major barriers to the application of CAM [3, 15, 17, 19]. Therefore, if improvement was made in technology, taste, and information spreading, CAM would become more popular.

## 4. Discussion

CAM, a distinct medical system, has developed for over 5000 years and played a great role in health care in China [10]. To date, this is the largest comprehensive study targeting at the students' attitude toward CAM in China. Most of our study population had a positive attitude toward CAM, which is similar to the results described in other counties [4, 20–24].

In our study, the CHBQ overall mean score was 48.87 ± 8.594, which was higher than that in other countries [1, 15, 25]. Participants of MU had a higher score than those of NU (49.32 ± 9.23 vs. 48.02 ± 7.08, *p* < 0.001). Students of TCM tended to hold a more positive attitude than those of WM (53.50 ± 7.405 vs. 45.58 ± 10.807, *p* < 0.001), which proves that proportion of CAM courses and self-application could lead to a difference in attitude. The CHBQ mean score became higher as the proportion of CAM courses increased, and the self-application of CAM was significantly associated with the CHBQ score (*p* < 0.001). Students with a more positive attitude toward CAM were more likely to recommend and more willing to learn CAM courses (*p* < 0.001). The results indicate that familiarity was highly linked to the use of CAM with increasing odds ratios, which is in line with the study of the University of California Irvine [14].

To our surprise, the most common source for students of WM to obtain CAM information was media (39.3%) rather than teachers (32.1%), which is similar to the results of studies in other counties [10, 17]. 28.3% WM students did not desire to learn more about CAM, which was higher than the average percentage (17.7%). The CHBQ overall mean score of WM students was the lowest among all the participants, which could be explained by the following reasons: Although CAM courses were compulsory, they only accounted for less than 10% in the curriculum of WM. Understanding the complicated CAM theory within the limit didactic hours was challenging for WM students [10, 26]. Another reason is the lack of “experiential/practical training.” A lot of the literature has reported that students needed “better integration of theory and practice” in CAM courses [10].

In our study, the female students held a more positive attitude toward CAM than the male students. Junior students were more positive than senior students (50.07 ± 7.642 vs 47.59 ± 9.34, *p* < 0.001), which is consistent with the investigation in other countries [12, 25, 27, 28].

The lower grade students had a stronger desire to learn CAM than higher grade students (89% vs 83%, *p* < 0.05). About 72.3% of the students who had never attended CAM courses wanted to learn CAM (*p* < 0.05), and 55.5% were willing to recommend CAM to others. The more positive attitude they had, the more likely they would recommend CAM [21].

Among the popular therapeutic methods, including acupuncture, herbology, food therapy, Tui Na (Chinese massage), cupping, moxibustion, Qi Gong, and Tai Chi [29], the students were more interested in acupuncture (50.7%), chiropractic (48.6%), TCM (47.3%), yoga (38.5%), meditation (28.8%), and Tai Chi (26.3%).

The major barrier for students to use CAM included “too time-consuming and bad tastes” (33.6%), “Western medicine was enough” (32.9%), and “lack of knowledge of CAM” (22.7%). In the survey, 79.1% of students agreed that CAM should be integrated into the curriculum of Western medicine. Meanwhile, nearly two-thirds (65%) of participants agreed that clinical doctors should equip themselves with CAM. And 83.5 % disagreed that CAM was a threat to public health.

Some researchers found that CAM courses had positive effects on the students' personal health in terms of physical exercise, better sleep, stress management, and decreased alcohol use [28, 30]. The students' willingness to learn CAM suggests the need for CAM courses in the university curriculum.

There are several limitations in the present study. The survey was based on self-reported data, so the recall bias might have affected the results. Although the overall response rate was 94%, some students were less familiar with CAM, which could also affect some results. In addition, multiple factors, such as race, educational and cultural background, and parents' profession, might contribute to the differences in attitude toward CAM between the groups. Besides, the survey was conducted in a particular city, and the results might differ in other regions.

## 5. Conclusions

In this study, the CHBQ overall mean score indicated that most undergraduates had a positive attitude toward CAM. A majority of students hoped to incorporate CAM courses into the curriculum or learn more about CAM. Also, 72.3% of those who had not attended CAM courses wanted to know more about CAM. The lower grade students had a stronger desire to learn than higher grade student. “Too time-consuming and bad tastes,” “Western medicine was enough,” and “lack of relevant knowledge” were found to be the three main interruptions in the use of CAM. The students' attitudes were in line with their willingness to recommend CAM to others. It is advisable to introduce or increase CAM courses in the college curriculum, and these courses may start in the lower grades. We hope this study can provide evidence for the authority in China to make appropriate changes and integrate CAM into the college curriculum, which will contribute to the overall health of the Chinese people.

## Figures and Tables

**Table 1:** Characteristics in MU and NU.

		MU (1448), Mean ± SD, 49.36 ± 9.226, *N* (%)	NU (706) Mean ± SD, 47.86 ± 7.023, *N* (%)	*p* value
Gender	Males	380 (26.2)	395 (55.9)	*p* < 0.001
	Females	1068 (73.8)	311 (44.1)	

Grade	First- and second-year classes	763 (52.7)	346 (49.0)	0.108
	Third-, fourth-, and fifth-year classes	685 (47.3)	360 (51.0)	

Age (years)	18–21	1163 (80.3)	551 (78.0)	0.219
	22–25	285 (19.7)	155 (22.0)	

Place of residency	Rural	680 (47.0)	336 (47.6)	0.783
	Urban	768 (53.0)	370 (52.4)	

Father or mother's level of education	＜High school	1057 (73.0)	513 (72.7)	0.870
	≥High school	391 (26.0)	193 (27.3)	

Proportion of CAM courses	0	129 (8.9)	337 (47.7)	*p* < 0.001
	1–10%	488 (33.7)	137 (19.4)	
	11%–20%	181 (12.5)	97 (13.7)	
	21%–30%	174 (12.0)	57 (8.1)	
	31%–40%	142 (9.8)	30 (4.2)	
	41%–50%	141 (9.7)	23 (3.3)	
	≥51%	193 (7.8)	25 (3.5)	

Source of information	Teacher	704 (48.6)	84 (11.9)	*p* < 0.001
	Books, newspapers, or magazines	264 (18.2)	214 (30.3)	
	Media or Internet	304 (21)	292 (41.4)	
	Others	176 (12.2)	116 (16.4)	

Self-application	Yes	895 (61.8)	395 (55.9)	*p* < 0.05
	No	553 (38.2)	311 (44.1)	

Hope to incorporate CAM into the curricula and learn more them	Yes	1270 (87.7)	584 (82.7)	*p* < 0.05
	No	178 (12.3)	122 (17.3)	

Publicity for CAM in place of residence	Bad	357 (24.7)	170 (24.1)	0.589
	Neutral	903 (62.4)	454 (64.3)	
	Good	188 (13.0)	82 (11.6)	

Willingness to recommend to others	Negative	94 (6.50)	44 (6.23)	*p* < 0.001
	Neutral	473 (32.6)	292 (41.36)	
	Positive	881 (60.9)	370 (52.41)	

MU = students of medical universities, NU = students of nonmedical universities. *p* < 0.05 was considered as significant. The mean CHBQ score was 49.49 ± 10.087 in all medical students (Table 2). Students of TCM have a more positive attitude than students of WM (53.50 ± 7.405 vs 45.58 ± 10.807, *p* < 0.001). Except for gender, the proportion of CAM courses in the curriculum, source of information, self-use, and publicity for CAM in place of residence were the factors that caused the differences in attitude. However, there was no significant difference in grade, age, place of residence, and father or mother's level of education. Our result indicated that education plays a vital role on the attitude toward CAM, which is similar to the findings of studies in other counties [14]. We also found that only 19.1% of TCM students thought the publicity for CAM in place of residence was good. We further analyzed the attitude score and the proportion of CAM courses in the curriculum.

**Table 2:** Characteristics in TCM and WM.

		TCM (*N* = 408), Mean ± SD, 53.50 ± 7.405, *N* (%)	WM (*N* = 417), Mean ± SD, 45.58 ± 10.807, *N* (%)	*p* Value
Gender	Males	101 (24.8)	176 (42.2)	*p* < 0.001
	Females	307 (75.2)	241 (57.8)	

Grade	First- and second-year class	122 (29.9)	119 (28.5)	0.666
	Third-, fourth-, and fifth-year class	286 (70.1)	298 (71.5)	

Age (years)	18–21	312 (76.5)	325 (77.9)	0.615
	22–25	96 (23.5)	92 (22.1)	

Place of residency	Rural	170 (41.7)	190 (45.6)	0.259
	Urban	238 (58.3)	227 (54.4)	

Father or mother's level of education	＜High school	279 (68.4)	277 (66.4)	0.549
	≥High school	129 (31.6)	140 (33.6)	

Proportion of CAM courses	0	N/A	58 (13.9)	*p* < 0.001
	1%–10%	N/A	287 (68.8)	
	11%–20%	N/A	41 (9.8)	
	21%–30%	34 (8.3)	31 (7.4)	
	31%–40%	65 (15.9)	N/A	
	41%–50%	95 (23.3)	N/A	
	≥51%	214 (52.5)	N/A	

Source of information	Teacher	372 (91.2)	134 (32.1)	*p* < 0.001
	Books, newspapers, or magazines	29 (7.1)	102 (24.5)	
	Media and Internet	7 (1.7)	164 (39.3)	
	Others	0 (0)	17 (4.0)	

Self-application	Yes	294 (72.1)	247 (59.2)	*p* < 0.001
	No	114 (27.9)	170 (40.8)	

Hope to incorporate CAM into the curricula and learn more them	Yes	386 (94.6)	299 (71.7)	*p* < 0.001
	No	22 (5.4)	118 (28.3)	

Publicity for CAM in place of residence	Bad	86 (21.1)	145 (34.8)	*p* < 0.001
	Neutral	244 (59.8)	254 (60.9)	
	Good	78 (19.1)	18 (4.3)	

Willingness to recommend to others	Negative	1 (0.2)	15 (3.6)	*p* < 0.001
	Neutral	39 (9.6)	256 (61.4)	
	Positive	368 (90.2)	146 (35.0)	

TCM = students of traditional Chinese medicine, WM = students of Western medicine. *p* < 0.05 was considered as significant.

**Table 3:** Beliefs and attitudes toward health and CAM.

	Disagree (%)	Neutral (%)	Agree (%)
Physical health and mental health are maintained by an underlying energy or vital force.	19.8	26.6	53.6
Health and disease are a reflection of balance between positive life-enhancing forces and negative destructive forces.	13.8	25.2	61.0
The body is essentially self-healing, and the task of a healthcare provider is to assist in the healing process.	29.4	24.5	46.1
A patient's symptoms should be regarded as a manifestation of a general imbalance or dysfunction affecting the whole body.	7.7	20.3	72
A patient's expectations, health beliefs, and values should be integrated into the patient care process.	4.3	13.6	82.1
Complementary therapies are a threat to public health.	83.5	9.4	7.1
Treatments not tested in a scientifically recognized manner should be discouraged.	36.8	23.6	39.6
Effects of complementary therapies are usually the result of a placebo effect.	73.9	15.8	10.3
Complementary therapies include ideas and methods from which conventional medicine could benefit.	8.3	20.5	71.2
Most complementary therapies stimulate the body's natural therapeutic powers.	19.7	30	50.3
I feel it is a common phenomenon that the people around my place of residence use CAM.	24.4	50.6	25
I think CAM should be integrated into WM.	3.9	17	79.1
I think CAM has fewer side effects than WM.	20	26	54
I think clinical doctors should be better equipped with knowledge of CAM.	7.7	27.2	65.1
I think all healthcare professional should accept that patients are using CAM.	3.4	21.6	75
I think CAM can only treat minor illnesses but not serious illnesses.	70.2	20.5	9.3
I would like to recommend CAM to others.	6.4	20	73.6

**Table 4:** Types of CAM the students wanted to study.

Types of CAM	Want to learn (*n*)	(%)
TCM	1019	47.3
Nutrition	943	43.8
Spirituality/prayer	222	10.3
Acupuncture	1047	48.6
Massage	1092	50.7
Tai Chi	567	26.3
Yoga	823	38.2
Homeopathy	168	7.8
Naturopathy	99	4.6
Meditation	620	28.8
Qi Gong	435	20.2
Osteopathic medicine	185	8.6
Ayurveda	54	2.5

## Data Availability

The data used to support the findings of this study are available from the corresponding author on request.
